# Perfluorinated Compounds as Test Media for Porous Membranes

**DOI:** 10.3390/membranes7030051

**Published:** 2017-09-05

**Authors:** Juliana I. Clodt, Volkan Filiz, Sergey Shishatskiy

**Affiliations:** Helmholtz-Zentrum Geesthacht, Institute of Polymer Research, Max-Planck-Str.1, 21502 Geesthacht, Germany; Juliana.Clodt@hzg.de (J.I.C.); volkan.filiz@hzg.de (V.F.)

**Keywords:** ultrafiltration, membrane characterization, perfluorinated compounds, porous membranes, permeance

## Abstract

We suggest a failure-free method of porous membranes characterization that gives the researcher the opportunity to compare and characterize properties of any porous membrane. This proposal is supported by an investigation of eight membranes made of different organic and inorganic materials, with nine different perfluorinated compounds. It was found that aromatic compounds, perfluorobenzene, and perfluorotoluene, used in the current study show properties different from other perfluorinated aliphatics. They demonstrate extreme deviation from the general sequence indicating the existence of π-π-interaction on the pore wall. The divergence of the flow for cyclic compounds from ideal e.g., linear compounds can be an indication of the pore dimension.

## 1. Introduction

Ultrafiltration (UF) membranes have attracted the increasing interests of scientists during the last decades. In the meantime, they are common in several applications as waste-water treatment [1,2,3], biomolecule separation [1,4], and controlled drug release [5]. UF membranes can be made from organic and inorganic materials by different techniques suitable for the material nature including casting, phase inversion, track-etching, anodizing, sintering, and film-stretching as conventional examples. Since each membrane is limited in their separation performance [6,7,8], new methods for the formation of UF membranes, especially of isoporous membranes [9], will be still present on the agenda of membrane scientists.

**Figure 1:**
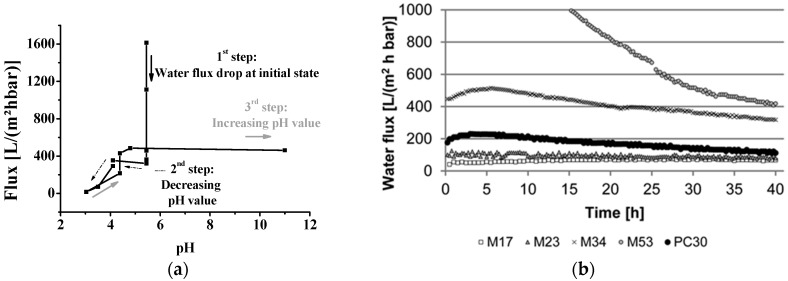
(**a**) pH-dependent water flux measurement of an isoporous block copolymer membrane with flux decline at initial state (water flux drop). (Reproduced from [11] with permission; Copyright 2012 Wiley-VCH Verlag GmbH & Co., KGaA). (**b**) Water flux measurements for membranes made from polystyrene-*block*-poly(4-vinylpyridine) with different pore sizes and with different flux decline. (Reproduced from [20] with permission; Copyright 2014 Royal Society of Chemistry).

For many years the only type of commercially available isoporous membranes was track-etched membranes. One success story to implement other methods to fabricate isoporous membranes starting ten years ago is the combination of self-assembly of amphiphilic block copolymers (S), and the non-solvent induced phase separation process (NIPS) called SNIPS [10]. With this method, UF membranes from various block copolymers can be prepared in a fast one-step process, leading to a new type of integral-asymmetric membrane with highly ordered, hexagonally arranged pores on the surface [11,12,13,14]. During the last few years, significant advances towards mass production of isoporous structures based on amphiphilic polymers has been achieved [15,16,17]. Nevertheless, the characterization of such membranes will cause problems if swellable polymer blocks are implemented and a standard solvent, e.g., water, will be used. In consequence, flux decline can occur as shown in Figure 1 [11,18,19,20,21].

Other problems for the characterization of UF membranes using water as liquid include fouling [22,23,24] and the unstable flow at different pressures [20]. The quality of water also plays an important role since deionized water made with different techniques, general tap water, or UV-treated water are used in different laboratories, dependent on the application and available facilities. The water flux values for UF membranes given in the literature are measured so far with several techniques, involving different: Pressures, time to stabilize the system until the value is measured, temperature, pre-treatment of the membranes in case of hydrophobic material, and so on.

In order to solve the aforementioned problems, one important question arises: How can we characterize UF membranes and get comparable data independent of the membranes’ chemical composition? The probing liquid which is inert for as many membrane materials as possible is needed, as well as an easy to implement method of the membranes permeance measurement. From the variety of liquid compounds available on the market, perfluorinated (PF) compounds were found to be the most promising. PF compounds are known for their very limited interaction/affinity with/to substances not containing fluorine, offering low surface tension, high density, and the wide variation in dynamic viscosity. In this work, PF compounds were chosen as a test media for the characterization of UF membranes of various nature. The PF compounds were chosen to cover as big a range of viscosity as possible, and included linear, cyclic and aromatic compounds. As examples of commercial membranes, ceramic anodic alumina, polymeric track-etched, and standard ultrafiltration membranes with isotropic and anisotropic morphology were chosen.

The results of our experiments confirm that porous membranes of almost any nature can be tested using PF liquids, and membrane properties can be compared. The membranes do not swell during the experiments and are not altered by PF compounds.

## 2. Results and Discussion

In this work, we want to justify the method suitable for standard membrane characterization with examples of membranes of different nature, cross-sectional morphology, and membrane preparation method: Polymeric membranes prepared from homopolymers by the phase inversion process and resulting in a broad pore size distribution, two mostly isoporous membranes: Polymeric track-etched and inorganic anodic alumina and highly isoporous membrane prepared from amphiphilic diblock copolymer manufactured by SNIPS. The PF compounds used for the membrane characterization have a carbon backbone of different arrangements, which allows for investigating the dependence of the fluid flow through the pores on the shape of the fluid molecule.

The first section will be dedicated to the verification of the method of permeance measurements with a water sensitive membrane and PF hexane. Later, we apply the method for the characterization of different membranes with various PF compounds. 

### 2.1. Verification of the Experimental Method for Sensitive Membranes

Since block copolymer membranes (BCPM) are the most sensitive of all membranes under investigation in this work, BCPM will be used to verify the method by using PF hexane for flux measurements. We chose one of the most common in-house made isoporous block copolymer membrane system, membranes made from polystyrene-*b*-poly(4-vinylpyridine) (PS-*b*-P4VP) with 4-vinylpyridine as a water swellable block.

**Figure 2:**
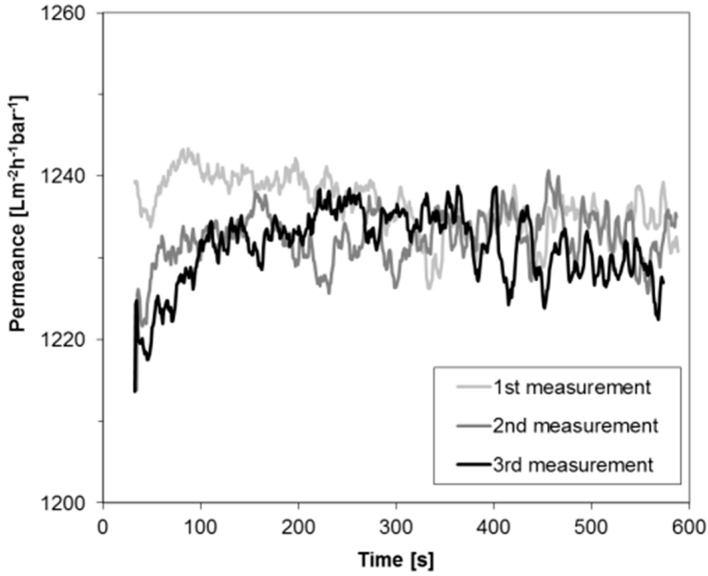
Permeance of PF hexane through the block copolymer membranes (BCPM) of PS_82.8_-*b*-P4VP_17.2_^190k^.

Figure 2 depicts the permeance of PF hexane for a BCPM made from PS_82.8_-*b*-P4VP_17.2_^190k^ diblock copolymer. One membrane stamp was measured three times in a row using the same PF hexane in order to evaluate the stability of the permeance which was found to be similar for all three measurements. Only a small deviation of less than 2% was found coming from the accuracy of the measurement collecting the data each second. Compared to water flux measurements, where big flux declines can be found at the beginning of the measurements (compare to Figure 1), this is an improvement for the characterization of performances of BCPM made from copolymers containing water swellable blocks. Deviation of the permeances at the beginning of the measurement may occur due to the wetting of the membrane and the instability of the pressure when the device is set on.

**Figure 3:**
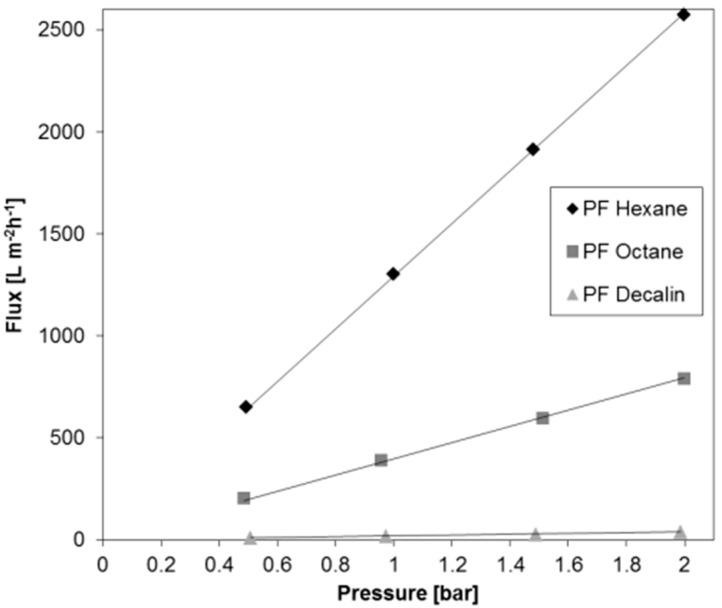
Flux through the BCPM of three perfluorinated (PF) compounds of various viscosity plotted against the trans-membrane pressure.

For the purpose of examining the stability of permeances depending on the transmembrane pressure, measurements were carried out at different pressures between 0.5 and 2 bar, i.e., within the design pressure range of the flux measurement facility. Figure 3 depicts the flux of three different PF compounds through the BCPM examined for the permeance stability (Figure 2) as a function of a trans-membrane pressure. For three PF compounds chosen to cover the full range of dynamic viscosity the permeance increases linearly, with the pressure indicating that in the selected pressure range any pressure suitable for the measurement can be chosen in dependence on the liquid viscosity.

### 2.2. Permeance of Ultrafiltration Membranes

In general, the theoretical volume flow rates of membranes may be calculated by the Equation (1) of Hagen-Poiseuille for a laminar flow in a simple straight cylinder:

(1) $$\mathrm{Volume}\;\mathrm{flow}\;\mathrm{rate}\;=\frac{\;\pi r^{4}\Delta p}{8\eta L}$$

where *r* is the radius of the open pore, Δ*p* is the transmembrane pressure, η is the dynamic viscosity of a liquid, and *L* the length of the cylindrical pore. Assuming that the *r* and *L* will be constant if one membrane sample is used for experiments with different liquids, and Δ*p* is kept constant through the experiment, the volume flow rate normalized to the transmembrane pressure, here labeled as permeance, is proportional to the reciprocal dynamic viscosity (See Equation (2)):

(2) $$\mathrm{Permeance}\;\sim \frac{\;1}{\eta }$$

**Figure 4:**
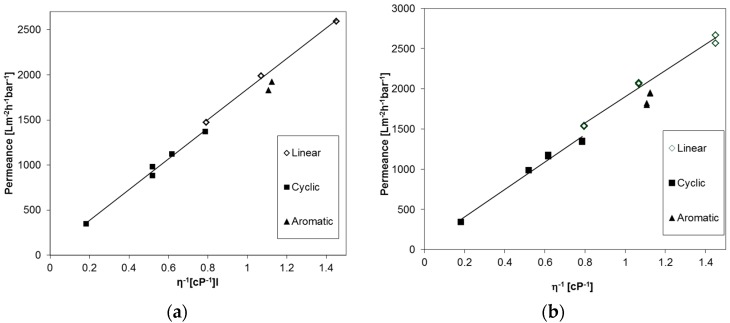
Permeances of Anodisc^®^ membrane with 20 nm pore diameter (**a**) and PE membrane with 50 nm pore diameter (**b**) against reciprocal dynamic viscosity of linear, cyclic aliphatic and aromatic PF compounds.

The permeances of different linear aliphatic, cyclic aliphatic, and aromatic PF compounds were studied for an anodic alumina membrane, Anodisc^®^ (GE Healthcare, Chalfont St Giles, UK) with 20 nm pore diameter, and a UPZP05205 polyethylene membrane (Millipore, Billerica, MA, USA) in order to prove applicability of the Equation (2) for PF compounds in general. The results are depicted in Figure 4 and at first sight a linear trend can be seen for both types of membranes, as expected. On the other hand, a small difference in the flow of molecules with different shapes is apparent. The permeance of aromatic PF compounds is lower than those of the linear aliphatic compounds, and additionally, a discontinuity in the trend of the linear and cyclic compounds appears.

**Figure 5:**
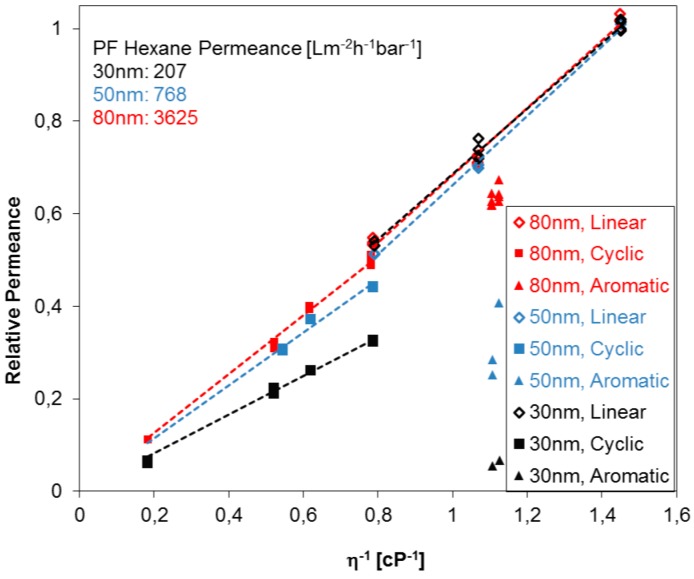
Relative permeance (dimensionless) of PC track-etched membranes with 30, 50 and 80 nm pore diameters against reciprocal dynamic viscosity of linear, cyclic, and aromatic PF compounds.

In order to explore further the break in the Hagen-Poiseuille trend for permeances found for the compounds with different molecule shapes, three PC track-etched membranes with different pore sizes were investigated. Figure 5 summarizes the relative permeances of different PF compounds for the case of three PC track-etched membranes (pore diameters 30, 50 and 80 nm) in dependence of the reciprocal viscosity. The relative permeance is shown in the graph where the highest permeance of PF hexane for each membrane is set to unity, and the others are set in proportion to it. The following tendencies were observed: Linear aliphatic as well as cyclic aliphatic PF compounds indicate each a proportional relative permeance to the reciprocal viscosity, as expected from Equation (2). However, track-etched PC membranes show considerable differences of relative permeances of PF compounds depending on the shape of the PF molecule. The permeances of cyclic aliphatic PF compounds, and especially aromatic PF compounds, are significantly smaller than the permeances of linear PF aliphatics, indicating, most probably, that the formation of immobilized molecular layers on the pore walls. Linear compounds are more flexible, and additionally, can be more easily oriented in the direction of the liquid flow, while in the case of cyclic compounds, steric hindrance may play an additional role leading to more distinctive immobilization on the pore wall. Immobilization of the molecules on the pore wall can cause a significant change of the permeance since the pore diameter of the studied membranes is comparable to the characteristic size of the molecule [25]. This trend increases with the decreasing pore diameter of the PC track-etched membranes and could further be used to determine the pore size without microscopy studies. Very small or neglectable differences of relative permeances between cyclic and linear PF compounds one indicate that the pore sizes are below 30 nm for PC track-etched membranes. This behavior could be implemented for other membrane materials as well, especially membranes with isoporosity, probably dependent on the materials nature and substructure. Aromatic compounds undergo immobilization more readily than aliphatic compounds since they have a tendency of π-π-interaction, which can be induced by the shear force near (in molecular size terms) the pore wall where the flow velocity is minimal [25]. Since PC track-etched membranes are, in general, made from polycarbonates with an aromatic backbone, π-π-interaction with the pore wall itself will also occur especially for track-etched membranes containing straight cylindrical pores.

**Figure 6:**
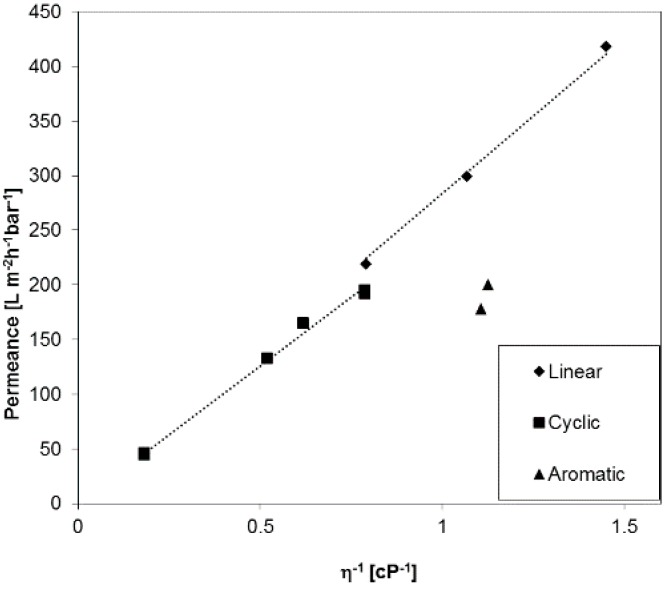
Permeance of a PAN membrane against reciprocal viscosity of linear, cyclic, and aromatic PF compounds.

In order to investigate liquid permeances for phase inversion membranes with broader pore size distribution as compared to the membranes discussed before, a PAN (polyacrylonitrile) ultrafiltration anisotropic membrane was selected exemplarily, and the results are depicted in Figure 6. The permeance for aromatic PF compounds has the same trend as we discussed for PC track-etched and the Anodisc^®^ membranes before. Interestingly, the gap between linear and cyclic PF compounds is lower than for the PC30, even though the PAN membrane has a smaller average pore diameter of 22 nm. For this type of membranes, the aforementioned influence of substructure may play an additional role for the immobilization of PF molecules on pore or substructural walls. The porosity of PAN membranes is much higher than for track-etched membranes, 12.4% as compared with 0.4% for PC30, and the PAN membranes have a wide pore size distribution that plays an important role [26].

**Figure 7:**
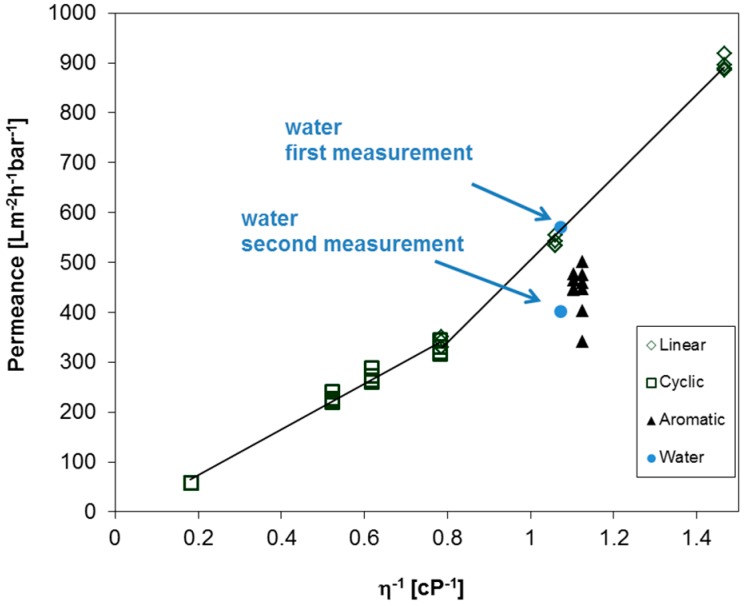
Permeances of BCPM made from PS_82.8_-*b*-P4VP_17.2_^190k^ against reciprocal viscosity of linear, cyclic, and aromatic PF compounds, respectively, and water. Each point represents the average value for one experiment with a liquid.

The question of whether the observed behavior of aromatic, cyclic, and linear PF compounds can be found for the aforementioned BCPM arises. Therefore, the permeances were measured for the set of different PF compounds and water, as depicted in Figure 7. Each compound was measured at least two times in a row to ensure the reproducibility of the obtained results. Similar to the other polymeric membranes used in this work, the permeance of aromatic PF compounds is lower as compared to aliphatic compounds. Interestingly, the permeances of cyclic aliphatic are again lower when compared to linear aliphatic compounds, but in this case the corresponding slopes overlap around 0.8 cP^−1^, and a steeper slope is observed for linear compounds as compared to the slope of cyclic compound without a gap of the trend line between linear and cyclic compounds. Furthermore, the trend for linear PF compounds unexpectedly does not approach zero at infinite dynamic viscosity, a fact to be examined further. On the one hand the average pore diameter of this BCPM is 42 nm, which should lead only to small differences in the pemeances of linear and cyclic compounds as observed for PC track-etched membranes discussed above. On the other hand, the substructure of BCPM plays an important role in their performances, as studied before [26]. Nevertheless, permeance measurements of PF compound could help to understand the behavior of BCPM, and should be studied in detail in future works depending on the pore sizes and substructure. In case of water, the flux decreases to about 30% already during the second measurement (compare first and second measurement in Figure 7), leading to further problems when the performance of such membranes is under investigation.

In order to compare the experimental and theoretical fluxes, the theoretical fluxes were calculated according to Hagen-Poiseulle’s law as follows, taking the porosities of the membranes into account (See Equation (2)):

(3) $$\mathrm{Theoretical}\;\mathrm{water}\;\mathrm{fluxes}\;=\;\frac{\;\pi r^{4}\Delta p\mathrm{Porosity}}{8\eta LA\Delta p}=\;\frac{\;r^{2}\mathrm{Porosity}}{8\eta L}$$

where *A* is the area of the pore corresponding *A =* π*r*^2^, *L* the length of the cylindrical pore, and the average surface porosity values were used as listed in Table 1. The tortuosity of the membrane is not taken into account since it is impossible to obtain the reliable value from any source of information either flow experiment or microscopy. Theoretical fluxes were calculated according to Equation (3) for the track-etched membranes, PC30, PC50, and PC80, which should have straight cylindrical pores through the whole membrane thickness. Surprisingly, theoretical permeances for PF hexane are around ten times lower than the experimental fluxes, namely theoretical fluxes 350, 80, and 10 L/m^−2^·h^−1^·bar^−1^ as compared to the experimental fluxes 3685, 775, and 211 L/m^−2^·h^−1^·bar^−1^ for PC80, PC50, and PC30. The calculation was based on the pore diameter of the entrance of the pore given by the supplier, and with the assumption that track-etched membranes have straight cylindrical pores perpendicular to the membrane surface through the whole thickness of the membrane. For track-etched membranes, the surface pore size is smaller than in the bulk of a membrane [27]. On the other hand, double and multiple pores having an origin in the overlapping of tracks of particles used for the polymer bombardment are often found on the membrane surface of the track-etched membranes [28]. Both of the facts are leading to higher experimental fluxes than the calculated one. For example, for the PC50 membrane, the theoretical, and experimental permeances would match each other when the average pore diameter is 88 nm.

## 3. Materials and Methods

### 3.1. Membranes Used in This Work

Polyacrylonitrile membranes were prepared by the phase inversion process [32]. Commercial polycarbonate track-etched membranes were purchased from Pieper (Germany): Supplier number PCN8CP04700, pore size 80 nm, thickness 6 µm, typical water flow rate 2 mL/(cm^2^·min) at 0.67 bar (in this work labelled as PC80); supplier number PCN5CP04700, pore size 50 nm, thickness 6 µm, typical water flow rate 1 mL/(cm^2^·min) at 0.67 bar (in this work labelled as PC50); supplier number PCN3CP04700 pore size 30 nm, thickness 6 µm, typical water flow rate 0.2 mL/(cm^2^·min) at 0.67 bar (in this work labelled as PC30); and, data as stated by the supplier. Polyethylene (PE) membranes, supplier number UPZP05205, pore size 50 nm were purchased from Millipore. An anodized alumina membrane Anodisc^®^ circle with support ring, 47 mm, 0.02 µm pore size was purchased from GE Healthcare GmbH. Isoporous diblock copolymer membranes (BCPM) used in this work were prepared according to a procedure published by Rangou et al. [12]. All membranes, their abbreviation used in this work, and their geometrical features are listed in Table 1.

### 3.2. Perfluorinated Compounds Used in This Work and Their Physical Data

The PF compounds used for the membrane performance measurements and their physical properties are listed in the Table 2. PF compounds were selected in order to analyze three different groups: Linear aliphatic compounds: Perfluorohexane, hexadecafluoroheptane, perfluorooctane; PF cyclic aliphatic compounds: Perfluoro(methylcyclohexane), perfluoro-1,3-dimethylcyclohexane, perfluorocycloether labeled as FC-77; and, aromatic compounds: Perfluorobenzene and perfluorotoluene. In order to compare the measurement data of the PF compounds with water, commonly used for flux measurements, demineralized water with an electrical conductivity of ≈0.055 μS·cm^−1^ was employed.

Dynamic viscosity was calculated from kinematic viscosity of the PF compounds, measured using a Lauda iVisc Viscometer Version 1.01 at 23 ± 1 °C in agreement with the temperature of the permeance measurement.

### 3.3. Liquid Permeance Measurement

**Figure 8:**
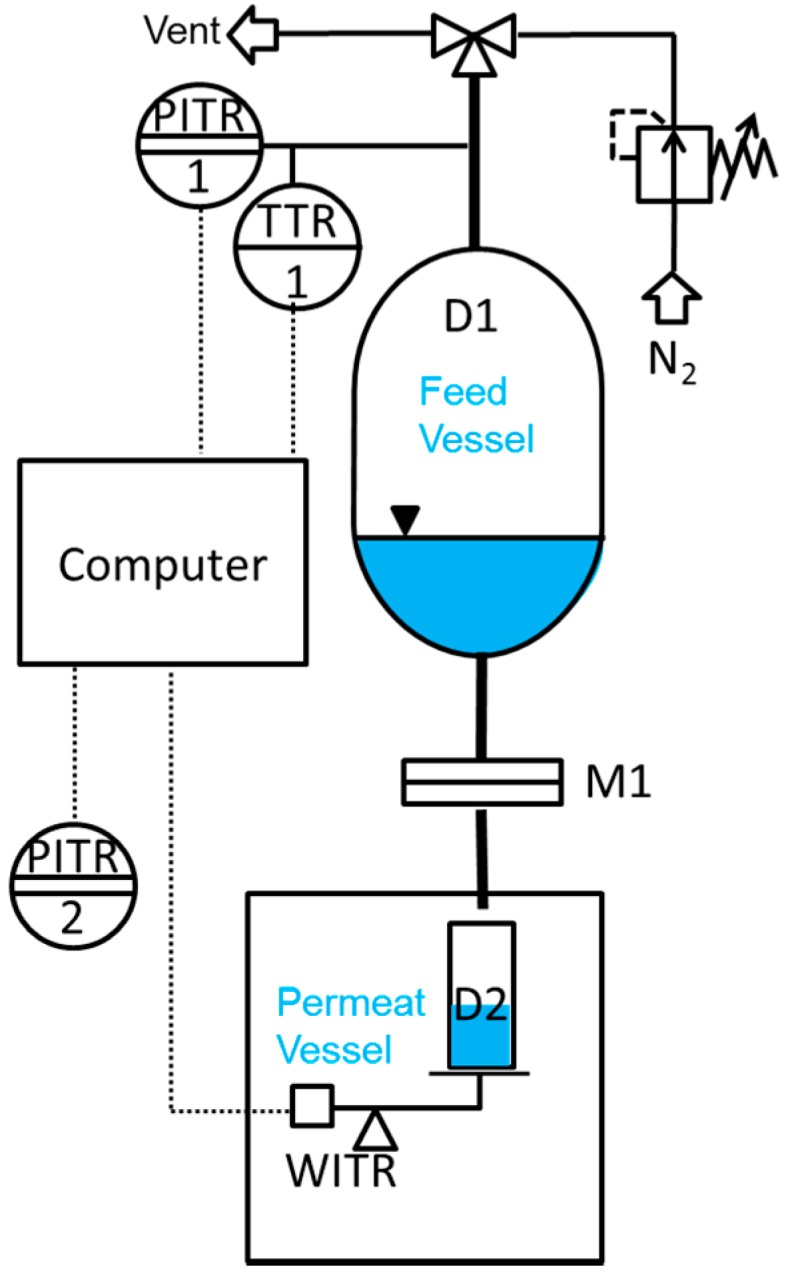
Scheme of the measurement facility for determination of liquid permeance for porous membranes.

Liquid permeance measurements were performed in a simple dead-end mode at room temperature using an in-house designed and manufactured testing facility, as schematically shown in Figure 8. A Millipore inline stainless steel filter with 20 mm sample diameter was used as a membrane sample holder. Nitrogen at relative pressures from 0.5 to 2.5 bar was used to create a driving force. The liquid flow rate through the membrane was measured gravimetrically every second by weight acquisition from the Mettler Toledo NewClassic MF MS1003S precision balance with 0.001 g accuracy. In our case, all data including pressure, temperature, and weight was transmitted to a computer for an easy evaluation.

The permeance (*P*) was calculated by normalizing the flux by the trans-membrane pressure (See Equation (4)):

(4) $$P=\frac{\;\Delta V}{A\;\Delta t\;\Delta p}$$

where Δ*V* is the volume of PF compound collected between two mass measurements, *A* is the membrane surface area, Δ*t* is the time between two mass measurements, and Δ*p* is the trans-membrane pressure.

The flux (*J*) through the membrane was calculated according to the Equation (5):

(5) $$J=\frac{\Delta V}{A\;\Delta t\;}$$

The trans-membrane pressure was varied in the range 0.5 to 2.5 bar depending on the PF liquid viscosity in order to ensure the collection of the experimental data during similar time intervals for all liquids. The independence of the flux on the trans-membrane pressure was studied and will be described further.

## 4. Conclusions

In this work we examined the use of PF compounds as possible universal media for the characterization of ultrafiltration membranes in order to avoid e.g., swelling of membranes in water, as discussed before [11,20]. Therefore, PF compounds of different molecular shape and electronic nature were studied. The following results were observed:

1. PF compounds can be used to study the permeances of sensitive membranes made from water or other solvent swellable materials.
2. Pressure dependent measurements show that the permeances of PF compounds are stable in the pressure difference range 0.5 to 2 bar, and the flux does not change significantly with time.
3. All studied PF aliphatic compounds, both linear and cyclic, show permeance gradually changing with the viscosity in full agreement with the Hagen–Poiseuille equation.
4. The Hagen–Poiseuille trends for linear and cyclic aliphatic compounds deviated increasingly with decreasing average pore diameter: The slope for the cyclic compounds is smaller than that for linear compounds and this observation may possibly be used for estimating the pore size of the membrane without microscopic study each time.
5. The permeances of aromatic PF compounds through ultrafiltration membranes made of materials of different nature are lower than the fluxes of aliphatic compounds presumably due to supramolecular interaction between aromatic PF molecules and pore walls.
6. Taking into account the considerations discussed above, one can conclude that most suitable PF compounds for the membrane characterization are perfluorooctane and FC-77 by 3M Company (St. Paul, MN, USA). These two compounds have very similar density and viscosity, while one is linear aliphatic and another cyclic ether.

## Figures and Tables

**Table 1:** Membranes and geometrical features used in this work.

Membrane	Abbr. Used in This Work	Average Pore Diameter (nm)	Porosity (%)
Polycarbonate track-etched PCN8CP04700	PC80	80 ^a^	2.0 ^a^
Polycarbonate track-etched PCN5CP04700	PC50	50 ^a^	1.2 ^a^
Polycarbonate track-etched PCN3CP04700	PC30	30 ^a^	0.4 ^a^
Polyethylene UPZP05205	PE50	50 ^a^	– ^c^
Anodized alumina Anodisc^®^	Anodisc	20 ^a^	40 ^b^
Polyacrylonitrile	PAN	22 ^b^	12.3 ^b^
Block copolymer membranes	BCPM	42 ^b^	29.5 ^b^

^a^ as stated by supplier; ^b^ calculated from SEM images as shown in previous work [12,29,30,31]; ^c^ accurate surface porosity is not possible to acquire from the SEM image due to very complex membrane morphology.

**Table 2:** Liquids used in this work and their physical data.

Substance	Abbreviation	*M*_w_^a^ (g/mol)	Density ^a^ (g/cm^−3^)	BP ^a^ (°C)	Dyn. Viscosity (cP) at 23 °C
Perfluorohexane	PF Hexane	339.04	1.686	59	0.690
Hexadecafluoroheptane	PF Heptane	388.05	1.731	83	0.936
Perfluorooctane	PF Octane	438.06	1.757	103	1.27
Perfluoro(methylcyclohexane)	PF MCH	350.05	1.784	76	1.62
Perfluoro-1,3-imethylcyclohexane	PF DMCH	400.06	1.838	101	1.93
Perfluorodecalin	PF Decalin	462.08	1.926	142	5.50
Perfluorocycloether	FC-77	416	1.767	97	1.27
Perfluorobenzene	PF Benzene	186.05	1.613	81	0.889
Perfluorotoluene	PF Toluene	236.06	1.664	104	0.903
Water	Water	18.02	0.995	100	0.932

^a^ The data as available by material safety data sheet or stated by a supplier.
